# Frequent chloroplast RNA editing in early-branching flowering plants: pilot studies on angiosperm-wide coexistence of editing sites and their nuclear specificity factors

**DOI:** 10.1186/s12862-016-0589-0

**Published:** 2016-01-25

**Authors:** Anke Hein, Monika Polsakiewicz, Volker Knoop

**Affiliations:** IZMB – Institut für Zelluläre und Molekulare Botanik, Abteilung Molekulare Evolution, Universität Bonn, Kirschallee 1, D-53115 Bonn, Germany

**Keywords:** *Amborella*, Pentatricopeptide repeat (PPR) proteins, Molecular phylogenetics, Pyrimidine exchange RNA editing, Mitochondria, Chloroplasts, RNA-binding proteins, Molecular coevolution

## Abstract

**Background:**

RNA editing by cytidine-to-uridine conversions is an essential step of RNA maturation in plant organelles. Some 30–50 sites of C-to-U RNA editing exist in chloroplasts of flowering plant models like *Arabidopsis*, rice or tobacco. We now predicted significantly more RNA editing in chloroplasts of early-branching angiosperm genera like *Amborella, Calycanthus, Ceratophyllum, Chloranthus, Illicium, Liriodendron, Magnolia*, *Nuphar* and *Zingiber*. Nuclear-encoded RNA-binding pentatricopeptide repeat (PPR) proteins are key editing factors expected to coevolve with their cognate RNA editing sites in the organelles.

**Results:**

With an extensive chloroplast transcriptome study we identified 138 sites of RNA editing in *Amborella trichopoda*, approximately the 3- to 4-fold of cp editing in *Arabidopsis thaliana* or *Oryza sativa*. Selected cDNA studies in the other early-branching flowering plant taxa furthermore reveal a high diversity of early angiosperm RNA editomes. Many of the now identified editing sites in *Amborella* have orthologues in ferns, lycophytes or hornworts. We investigated the evolution of CRR28 and RARE1, two known *Arabidopsis* RNA editing factors responsible for cp editing events ndhBeU467PL, ndhDeU878SL and accDeU794SL, respectively, all of which we now found conserved in *Amborella*. In a phylogenetically wide sampling of 65 angiosperm genomes we find evidence for only one single loss of CRR28 in chickpea but several independent losses of RARE1, perfectly congruent with the presence of their cognate editing sites in the respective cpDNAs.

**Conclusion:**

Chloroplast RNA editing is much more abundant in early-branching than in widely investigated model flowering plants. RNA editing specificity factors can be traced back for more than 120 million years of angiosperm evolution and show highly divergent patterns of evolutionary losses, matching the presence of their target editing events.

**Electronic supplementary material:**

The online version of this article (doi:10.1186/s12862-016-0589-0) contains supplementary material, which is available to authorized users.

## Background

The discovery of C-to-U RNA editing in plant mitochondria [1–3] was soon followed by a report on the same type of RNA editing also existing in chloroplasts [4]. This first reported case of chloroplast RNA editing creates an AUG methionine start codon in the maize *rpl2* mRNA by converting a genomically encoded ACG threonine codon. Coincidentally, the very first nuclear specificity factor (CRR4) identified for a plant organelle RNA editing event 14 years later [5] also affects the creation of a start codon, in this case the one in the *ndhD* mRNA of *Arabidopsis thaliana*. This and many other nuclear-encoded RNA editing factors affecting chloroplast or mitochondrial RNA editing events turned out to belong to a plant-specific subclade of RNA-binding pentatricopeptide repeat proteins [6–12]. These observations and the phylogenetic coexistence of chloroplast and mitochondrial RNA editing – both are present in all plant clades with the unique exception of complex-thalloid liverworts – suggest that the mechanisms of C-to-U RNA editing are essentially the same in the two endosymbiotic plant cell organelles [13].

One striking discrepancy of RNA editing in the two organelles, however, concerns the observed abundance of editing events in widely studied model plants such as *Arabidopsis thaliana*, *Nicotiana tabacum* or *Oryza sativa* where some 400–500 mitochondrial sites coexist with only 30–40 such sites in chloroplasts [14]. Interestingly, such a strong bias of mitochondrial vs. chloroplast RNA editing likewise exists in the moss *Physcomitrella patens* where only two events of chloroplast editing [15] are contrasted by eleven such sites in mitochondria [16]. Its low overall RNA editing frequencies have made *Physcomitrella* a particular interesting plant model to study RNA editing. In fact, the moss has recently become the first organism with a completed mutual assignment of organelle editing sites to their nuclear PPR protein cofactors [17–19].

The reasons for significantly higher frequencies of mitochondrial over chloroplast editing in plants remain unclear. Such a mitochondrial vs. chloroplast editing bias seems to disappear when organelle RNA editing ultimately reaches record frequencies, approaching or even exceeding 1,000 editing events per organelle. A new organelle RNA editing record with more than 3,500 editing sites in the chloroplasts of the lycophyte *Selaginella uncinata* [20] even exceeds a previous mitochondrial RNA editing record in *S. moellendorffii* [21].

Plant organelle RNA editing dominatingly serves to re-establish evolutionarily conserved amino acid codon identities in mRNAs. This feature makes this type of RNA editing predictable to a very reasonable extent. To this end, the PREPACT software has been developed, which in its version 2 allows to predict RNA editing for entire new organelle genomes based on a manually curated reference organelle trancriptome database [22, 23]. Using this PREPACT feature we predicted significantly more chloroplast RNA editing for the meantime available chloroplast genomes of early-branching, “basal” angiosperm genera than for the hitherto widely investigated model angiosperms such as *Arabidopsis, Nicotiana* or *Oryza*. Our subsequent cDNA analyses confirm these expectations of high diversities and frequencies of cp editing in the basal angiosperms. The observations suggest a tremendous decrease of chloroplast RNA editing frequencies during flowering plant evolution, in line with previous reports describing losses of mitochondrial RNA editing on different levels of angiosperm diversification [24–28].

The gain or loss of organelle RNA editing sites can be expected to be correlated with an accompanying gain or loss of nuclear RNA editing factors or, alternatively, a functional extension or restriction of editing factors acting on several editing sites simultaneously. We observed interesting cases of chloroplast RNA editing sites conserved between the basal angiosperm *Amborella* and the model angiosperm *Arabidopsis,* for which chloroplast editing site specificity factors have already been characterized. Selecting two cases for pilot studies we found evidence for multiple independent losses of editing factor RARE1 simultaneously with its cognate RNA editing site accDeU794SL among angiosperms. In stark contrast, we find evidence for only one single loss of editing factor CRR28 addressing two editing sites simultaneously (ndhBeU467PL and ndhDeU878SL), perfectly correlating with the serial loss of both editing sites exclusively in chickpea (*Cicer arietinum*).

## Results

### The *Amborella* chloroplast editome

Using PREPACT’s feature to predict RNA editing for entire organelle genomes we found predictions of up to more than one hundred RNA editing sites for the chloroplast genomes of several angiosperms representing early branches in the phylogeny of flowering plants such as *Amborella trichopoda, Calycanthus floridus, Ceratophyllum demersum, Chloranthus spicatus, Illicium oligandrum, Liriodendron tulipifera, Magnolia kwangsiensis, Nuphar advena* and *Zingiber spectabile*. For example, in the case of *Amborella trichopoda*, the only living representative of the likely sister lineage to all other extant flowering plants, we found predictions of 90, 142 or 162 sites of C-to-U RNA editing to restore conserved codons at stringency thresholds of 90, 80 or 70 %, respectively, of the 17 references implemented in PREPACT 2.0 (Additional file 1). An example of the PREPACT prognosis for the *Amborella trichopoda* chloroplast *ndhD* gene is shown in Additional file 2. These observations, suggestive of significantly more frequent chloroplast RNA editing at the origin of angiosperms, prompted us to extensively investigate chloroplast cDNAs in *Amborella trichopoda*, accompanied by selective cDNA analyses in the other early-branching flowering plant taxa.

**Fig. 1:**
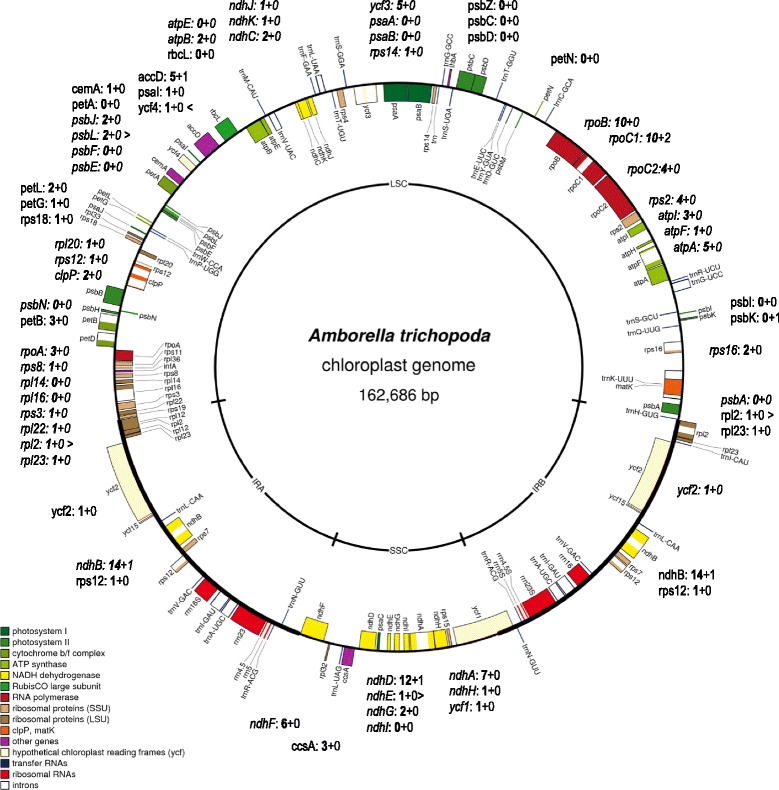
Map of the *Amborella trichopoda* plastome drawn with the OGDRAW tool [75]. Different colors indicate functional gene categories as indicated in the legend. Numbers of non-silent (bold) and silent (behind the plus symbol) C-to-U RNA-editing sites identified in the respective cDNAs are indicated next to each protein-coding gene. Italics indicate genes transcribed clockwise (inner circle) and normal font indicating genes transcribed counterclockwise (outer circle). Larger than (>) and smaller than (<) labels indicate the creation of start or stop codons by RNA editing, respectively

Altogether we identified 138 sites of C-to-U RNA editing (132 non-silent and 6 silent editing events) in 46 chloroplast mRNAs of *Amborella trichopoda* (Fig. 1). RNA editing prognosis turned out to be perfect for 29 of the chloroplast genes where RNA editing was predicted (Additional file 1). We additionally checked another 14 genes lacking predictions for RNA editing sites (*atpE, petA, petN, psaA, psaB, psbA, psbC, psbD, psbE, psbF, psbI, psbZ, rbcL* and *rpl14*) and could verify the absence of editing in these (Fig. 1). A total of 51 *Amborella* chloroplast editing events now identified had no reported counterparts in other angiosperms. Of those, however, 30 have counterparts in the reported editomes of the ferns *Adiantum* [29], *Ophioglossum* or *Psilotum* [30], the lycophyte *Selaginella* [20] or the hornwort *Anthoceros* [31] (Additional file 1).

Nineteen sites of RNA editing identified in our *Amborella* cDNA studies were unpredicted, comprising 13 codon-changing sites of editing below threshold levels plus six sites of silent editing (in *accD, ndhB, ndhD, psbK* and *rpoC1*). Particularly unexpected codon-changing “extra” edits are ccsAeU68TM and rpoBeU2926LF that cannot be explained from reference comparisons. It remains to be seen whether these are taxon-specific individual “orphan” edits that show up occasionally (such as chloroplast editing psbZeU50SL in *Arabidopsis*) or whether they are shared with at least some related taxa. *Vice versa*, we could not confirm 36 of the reasonably predicted chloroplast candidate RNA editing sites in *Amborella*. While a lack of editing at most of these sites may be explained by relaxed sequence conservation in other taxa, the lack of RNA editing events, rpl16eU310HY, rpoC1eU389SL, rpoC1eU617PL and rpoC1eU760LF, which have already been documented in other taxa, is surprising (Additional file 1).

### Highly variable editomes in early angiosperms

**Fig. 2:**
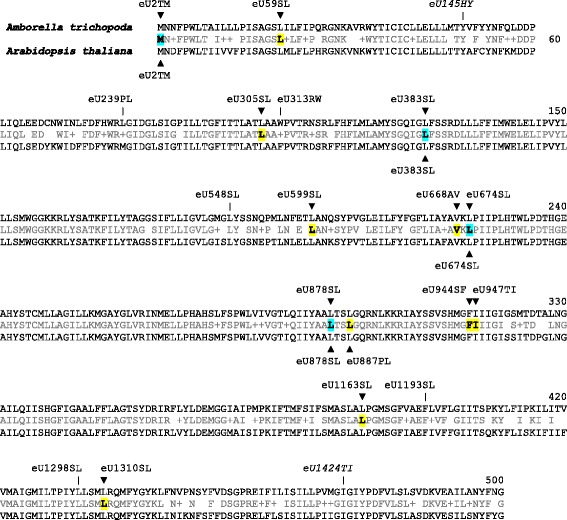
RNA editing in the chloroplast *ndhD* locus. Arrowheads indicate editing events in *Amborella trichopoda* (top) and *Arabidopsis thaliana* (bottom) reconstituting conserved amino acid identities (shaded, bold). Further editing events identified in other taxa (Table 1) are indicated by the pipe symbol (|) and likewise reconstitute conserved amino acids. Two further candidate edits ndhDeU145HY and ndhDeU1424TI (italics) in *Zingiber spectabile* remain to be investigated

An example, how RNA editing reconstitutes conserved codon identities is exemplarily shown for the heavily edited *ndhD* gene (Fig. 2). Only four of the twelve RNA editing events now confirmed in *ndhD* of *Amborella* are shared with *Arabidopsis*. Other than an overall higher amount of chloroplast RNA editing in the representatives of the ancient angiosperm lineages, we also found indications for highly variable patterns of RNA editing among them. Again, an example is shown for the highly edited *ndhD* locus (Table 1). On top of the twelve confirmed RNA editing events in *Amborella*, six additional editing sites were predicted and correctly confirmed in the other basal angiosperm taxa and two more, as yet unconfirmed editing sites (ndhDeU145HY and ndhDeU1424TI) may exist in *Zingiber spectabile*. Many more additional sites of RNA editing are predicted for the other basal angiosperm chloroplast genes, of which we could already verify more than 50, mainly in *Chloranthus* and *Illicium* (not shown). Once completed, the editomes of those taxa will become further additions to the PREPACT reference database. At present, it remains unclear to which extent the diversity of chloroplast RNA editing among the early angiosperms reflects independent gains or losses of editing in evolution. Should the latter dominate, one can assume the ancestral angiosperm chloroplast editome to comprise more than 200 RNA editing sites.

**Table 1:** RNA editing patterns in the chloroplast *ndhD* gene of the selected early branching angiosperms *Amborella trichopoda, Calycanthus floridus, Ceratophyllum demersum, Chloranthus spicatus, Illicium oligandrum, Liriodendron tulipifera*, *Magnolia kwangsiensis, Nuphar advena* and *Zingiber spectabile* in comparison to the ones of *Arabidopsis thaliana* and *Cucumis sativus*

	*Amborella trichopoda*	*Calycanthus floridus*	*Ceratophyllum demersum*	*Chloranthus spicatus*	*Illicium oligandrum*	*Liriodendron tulipifera*	*Magnolia kwangsiensis*	*Nuphar advena*	*Zingiber spectabile*	*Arabidopsis thaliana*	*Cucumis sativus*
eU2TM	**ed**	**ed**	ed	**ed**	**ed**	**ed**	**ed**	**ed**	ed	**ed**	**ed**
eU59SL	**ed**	**ed**	ed	**ed**	**ed**	**ed**	**ed**	p	C	p	p
*eU145HY*	p	p	p	p	p	p	p	p	ed	p	p
eU239PL	p	**ed**	p	p	p	p	p	p	p	M	p
eU305SL	**ed**	p	p	p	p	p	p	**ed**	p	p	p
eU313RW	p	**ed**	p	**ed**	p	**ed**	**ed**	**ed**	p	F	p
eU383SL	**ed**	**ed**	p	**ed**	**ed**	**ed**	**ed**	p	ed	**ed**	**ed**
eU548SL	p	**ed**	p	**ed**	**ed**	**ed**	**ed**	p	p	p	p
eU599SL	**ed**	p	ed	**ed**	p	**ed**	**ed**	p	p	p	**ed**
eU668AV	**ed**	p	p	p	**ed**	p	p	p	p	p	p
eU674SL*	**ed**	**ed**	p	**ed**	**ed**	**ed**	p	**ed**	p	**ed**	**ed**
eU878SL	**ed**	**ed**	ed	**ed**	**ed**	**ed**	**ed**	**ed**	ed	**ed**	**ed**
eU887PL*	p	p	p	**ed**	**ed**	**ed**	**ed**	p	ed	**ed**	**ed**
eU944SF	**ed**	p	p	p	p	p	p	p	p	p	p
eU947TI	**ed**	**ed**	p	p	p	**ed**	**ed**	p	ed	p	p
eU1163SL	**ed**	p	p	p	p	p	p	p	p	p	p
eU1193SL	F	p	p	**ed**	**ed**	**ed**	**ed**	p	ed	p	p
eU1298SL	p	**ed**	ed	**ed**	**ed**	**ed**	**ed**	**ed**	p	p	p
eU1310SL	**ed**	p	p	**ed**	**ed**	**ed**	**ed**	**ed**	p	p	p
*eU1424TI*	p	p	p	p	p	p	p	p	ed	p	p
**18** *+ 2*	**12**	**10**	5	**12**	**11**	**13**	**12**	**7**	8	**5**	**6**

A ‘p’ indicates a pre-edited status, i.e. a thymidine on DNA level. Editing nearly always restores universally conserved amino acids except for a cysteine (C) in *Zingiber* corresponding to ndhDeU59SL, a methionine (M) in *Arabidopsis* in position ndhDeU239PL and phenylalanines (F) in *Arabidopsis* in position ndhDeU313RW and in *Amborella* in position ndhDeU1193SL. Editing (ed in bold) has been investigated in this study except for *Ceratophyllum* and *Zingiber* (predictions only) and for *Arabidopsis* and *Cucumis* where it was analyzed previously. Editing events eU674SL and eU887PL labelled with asterisks may cause alternative codon changes (PL, SL, SF) in some taxa

### Updating previous angiosperm editome references

**Fig. 3:**
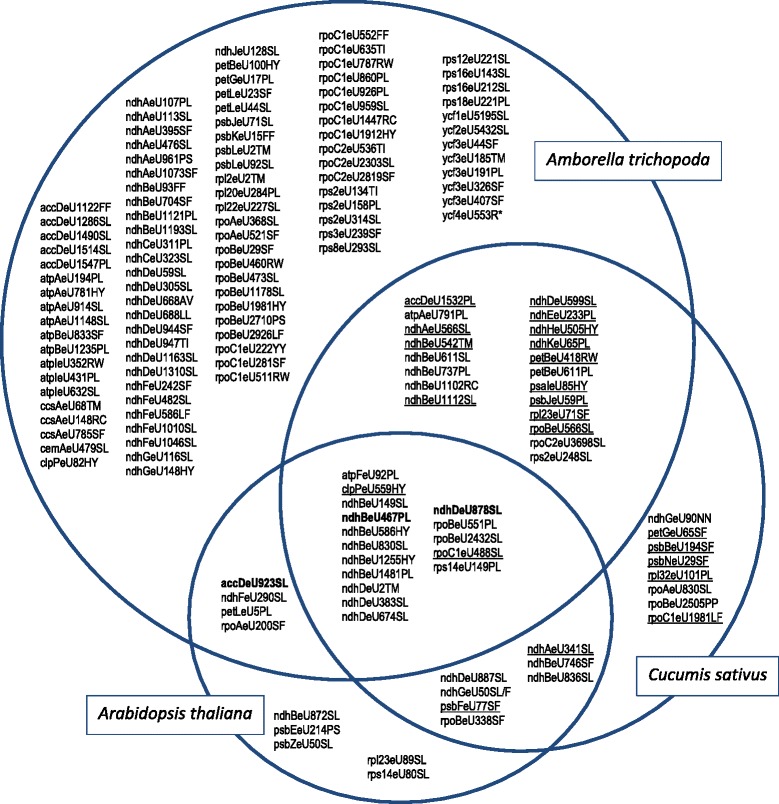
Venn diagram showing the occurrence of chloroplast RNA editing sites in *Amborella trichopoda, Arabidopsis thaliana* and *Cucumis sativus*. Editing position numbering refers to *Amborella* for shared sites. Underlining indicates RNA editing sites in *Cucumis* predicted and confirmed in the course of this study to extend the cucumber cp editome reported previously [32]. Highlighted in bold are sites ndhBeU467PL and ndhDeU878SL previously shown to be affected by editing factor CRR28 [37] and accDeU923SL, corresponding to *Arabidopsis thaliana* accDeU794SL, shown to be affected by editing factor RARE1 [39]

In the course of our studies we found reason to believe that the chloroplast editome of cucumber (*Cucumis sativus*) is substantially larger than previously reported [32]. Indeed, we were able to confirm an additional 22 of predicted sites of RNA editing in cucumber with our independent cDNA analyses (Fig. 3), underlining the value of the predictive approach to trigger reinspection of organelle transcriptome data. Because editing event ndhAeU341SL in *Cucumis* was among the now confirmed sites, we used the opportunity to also check upon the orthologous site in *Arabidopsis* on which conflicting reports had been published [33–35]. We could confirm editing ndhAeU341SL also in *Arabidopsis*, congruent with the 2001 report by Lutz and Maliga. We found editing petLeU5PL in *Amborella* reported previously [36] to be edited only very inefficiently (Additional file 1) It remains unclear as yet whether ecotype/isolate variance, cultivation conditions or the methods of cDNA analyses (see also Additional file 1) play a role for such conflicting observations. Of the now altogether 51 sites of RNA editing in the cucumber chloroplast editome, seven are exclusively shared with *Arabidopsis*, 20 exclusively with *Amborella* and 16 jointly with both these species (Fig. 3). Like the new *Amborella* chloroplast editome, the updated reference editomes of cucumber and *Arabidopsis* will be available with a soon forthcoming update of PREPACT (Lenz et al., in preparation).

### The coexistence of RNA editing sites and nuclear cofactors

The set of chloroplast editing sites shared between *Amborella trichopoda* and *Arabidopsis thaliana* (Fig. 3) includes RNA editing positions for which nuclear-encoded PPR proteins have already been identified as site-specificity factors in *Arabidopsis*. RNA editing events ndhBeU467PL and ndhDeU878SL in *Arabidopsis* are both affected by editing factor CRR28 [37, 38] whereas editing event accDeU794SL (corresponding to accDeU923SL in *Amborella*) is affected by RARE1 [39].

**Fig. 4:**
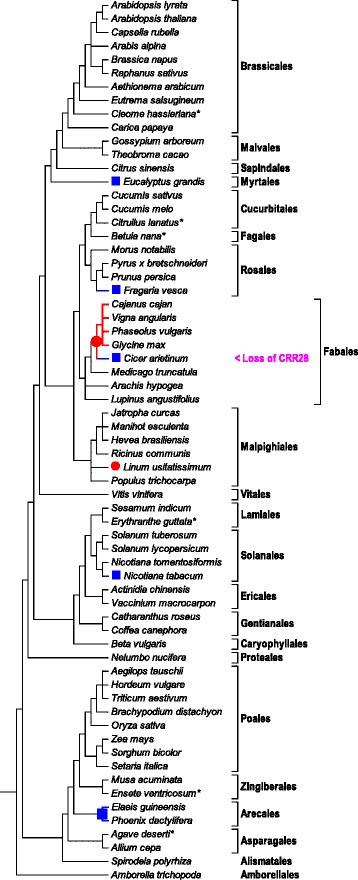
Cladogram of 65 selected angiosperms for which reliable protein model and/or genome data are available, based on current insights on angiosperm phylogeny with orders indicated. No chloroplast genome data are currently available for taxa marked with an asterisk and for *Cajanus cajan* (pigeon pea). The *ndhB* and *ndhD* genes of the latter have been analyzed individually in this study, however. Chloroplast editing sites ndhBeU467PL and ndhDeU878SL and their cognate specificity co-factor CRR28 are widely distributed among angiosperms except cases where labels are attached. Editing event ndhDeU878SL has been lost independently (filled squares) in *Eucalyptus, Fragaria, Cicer, Nicotiana* and in the palms (Arecales). Editing event ndhBeU467PL has been lost independently (filled circles) in *Linum* and in a subclade of Fabales including chickpea (*Cicer arietinum)*. CRR28 homologues are identified in all taxa (Additional file 3) except in chickpea where both editing sites are absent

The now observed conservation of editing events ndhBeU467PL and ndhDeU878SL not only between *Amborella, Arabidopsis* and *Cucumis* (Fig. 3) but also among the other basal angiosperms investigated (see e.g. Table 1 for ndhDeU878SL) suggests that CRR28 may be a very ancient editing specificity factor. Orthologues of CRR28 could hence be expected to be widely present in angiosperm genomes including the early-branching lineages. We screened available angiosperm sequence data focusing on species with high quality protein model or genome (and plastome) sequence data (see Materials and Methods), which ultimately resulted in a set of 65 angiosperm taxa of wide phylogenetic distribution (Fig. 4). CRR28 orthologues could be identified in 64 of those angiosperm genomes (Additional file 3). Although not offering significant phylogenetic resolution for deeper nodes, the molecular phylogeny of the flowering plant CRR28 orthologues agrees well with the independent current insights on angiosperm phylogeny (Fig. 4).

RNA editing site ndhDeU878SL is independently lost during angiosperm evolution at least five times: in *Eucalyptus*, in *Fragaria*, in *Cicer*, in *Nicotiana* and in the Arecales (Fig. 4). Conversely, loss of editing site ndhBeU467PL has occurred in *Linum* and in a “bean” subclade of Fabales including chickpea (*Cicer arietinum*). We identified unequivocal CRR28 orthologues in all taxa including those where the one or the other of the two editing sites is lost (Additional file 3). The only example of a taxon where no CRR28 orthologue could be identified is the *Cicer arietinum* genome, in perfect agreement with a secondary loss of the ndhDeU878SL editing site subsequent to the phylogenetically deeper loss of editing event ndhBeU467PL among the Fabales (Fig. 4). This suggests functional retention of CRR28 as long as at least one of the two editing sites needs to be served, but its quick disappearance once both editing sites are lost simultaneously.

To exclude potential cpDNA sequence errors and to investigate the chickpea case further we investigated two different *C. arietinum* varieties (chab I and nigrum) and the related *Cicer* species *C. pinnatifolium* for the *ndhB* and *ndhD* sites and could perfectly confirm the absence of both RNA editing sites in all three *Cicer* samples resulting from conversion of the affected cytidine positions into thymidines. Since no complete plastome sequence is hitherto available for *Cajanus cajan* (pigeon pea) we investigated the *ndhB* and *ndhD* sites individually on DNA and cDNA level. We observed loss of editing event ndhBeU467PL owing to a genomic C-to-T conversion but retention of the ndhDeU878SL editing event, hence exactly as in *Glycine, Phaseolus* and *Vigna* (Fig. 4).

**Fig. 5:**
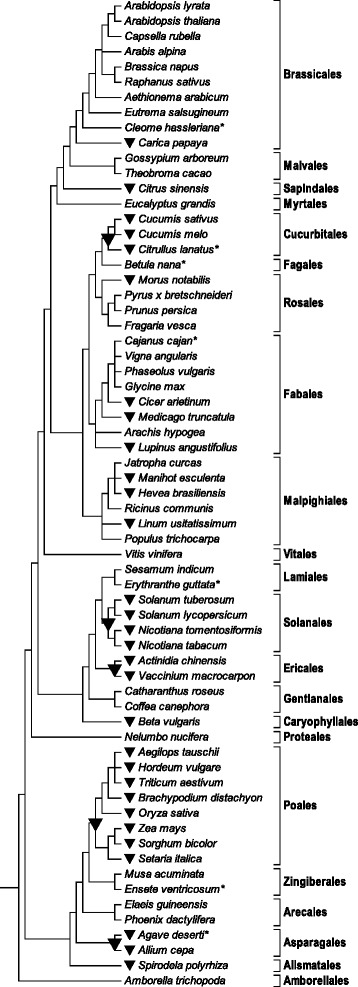
Cladogram of 65 flowering plants as in Fig. 4. Chloroplast RNA editing site accDeU923SL (or the *accD* gene altogether) is lost at least 14 times independently during angiosperm evolution (no plastome sequences are available for species marked with an asterisk). The loss of the accDeU923SL editing event is consistently accompanied by an apparent absence of RARE1 (black triangle) whereas RARE1 orthologues are always identified in the other taxa (Additional file 4)

An entirely different picture emerges for the case of RARE1 serving editing event accDeU794SL in *Arabidopsis thaliana* (corresponding to 923SL in *Amborella*). This editing is absent in 30 of the 65 sampled angiosperms, either owing to conversion to a genomic thymidine or by loss of the entire *accD* gene from the chloroplast DNAs altogether. In the light of the current insights on flowering plant phylogeny, this suggests at least 14 independent losses (Fig. 5). Whereas RARE1 orthologues can be confidently identified in all taxa requiring *accD* editing (Additional file 4), none can be identified in any of the 30 cases where the *accD* editing event is lost for the one or the other reason. Adding to the insight on CRR28 in the chickpea case this suggests a surprisingly fast disintegration of editing factors once their function becomes obsolete after loss of their cognate RNA editing site.

**Fig. 6:**
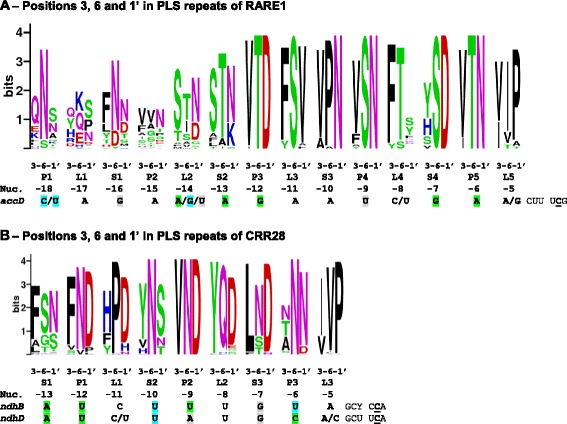
Matching key PPR residues with target sequences for RARE1 and CRR28. Relevant key residues in PPR positions 3, 6 and 1’ were selected from the total alignments of 35 RARE 1 (A) and 64 CRR28 (B) orthologues in angiosperms to obtain weblogo conservation plots at http://weblogo.berkeley.edu/logo.cgi [76]. Target sequences are aligned with the terminal S-type PPR (which contributes position 1’ to the preceding L-type PPR) juxtaposed with nucleotide -4 in front of the C-to-U editing site (underlined). Ambiguities are indicated where represented at least twice in the corresponding targets. Green shading indicates perfect matching of positions 6 and 1’ in P- and S-type repeats with corresponding nucleotides according to the proposed core binding code (T + N:A, N + N:C, T + D:G, N + D:U). Blue matching indicates distinction of pyrimidines vs. purines in position 6 only and grey shading indicates mismatches. Allowing for replacement of threonine by serine matches RARE1 PPR S4 (S + D) with G in its target and CRR28 (S + N) with A in its targets, but also mismatches RARE1 P4 (S + N) with U

CRR28 and RARE1 both are editing factors with a DYW domain. The likely key residues for deaminase activity and zinc coordination [40–42] and several other alignment positions in the carboxyterminal E and DYW domains are universally conserved in all 64 CRR28 and 35 RARE1 homologues in the angiosperms. Given the wide conservation of editing targets among the flowering plants we investigated conservation of the key PPR positions 6 and 1’ and the additionally proposed position 3 suggested as relevant for RNA binding [43, 44]. To this end, we created weblogo plots displaying conservation of those extracted positions for both proteins and aligned them with their chloroplast mRNA targets (Fig. 6). The PPR arrays of both proteins show only rather limited matches with their targets according to the proposed recognition code. The P- and S-type PPRs S3 and P4 in RARE1 show a striking conservation of non-canonical amino acids at the three positions supposedly relevant for RNA binding that are not yet accounted for. Particularly noteworthy is the strong conservation of L-type repeats L1, L2 and L3 in CRR28 and L3 and L5 in RARE1 given that L-type repeats have so far not been considered relevant to mediate RNA-binding. Most notably, the combination of a valine in position 6 and a proline in position 1’ is juxtaposed to adenines both in L3 of CRR28 and L5 of RARE1.

## Discussion

Here, we have shown significantly more chloroplast RNA editing to exist in early branching flowering plants as compared to the hitherto widely investigated angiosperm model species like e.g. *Arabidopsis,* tobacco or rice. This observation could either be explained by gradual losses of cp editing sites during the later diversification of flowering plants or by independent gains of editing in the basal angiosperm lineages. Very likely both factors contribute to the current situation in the extant angiosperms but an overall loss of RNA editing during flowering plant evolution appears to dominate [24–28, 45]. The 15 sites of RNA editing in *ndhD* alone that are shared between at least two early-branching angiosperms (Table 1) are likely examples for ancient RNA editing sites present early in the flowering plant stem lineage.

The plethora of chloroplast RNA editing initially predicted and subsequently confirmed in the basal angiosperms and the here presented case of many additional chloroplast RNA editing sites confirmed in *Cucumis sativus* underline the value of careful bioinformatic analyses of organelle genomes with tools such as PREPACT. We currently observe ever more examples of previously overlooked RNA editing events in published organelle editomes (unpublished observations). One key issue here may be the ever increasing use of RNA-seq and downstream bioinformatic pipelines that need careful adaptation to properly detect plant organelle RNA editing events. Editome information will continuously be updated with future versions of the references implemented in PREPACT (Henning Lenz, A.H, V.K., in preparation).

The presence of organelle RNA editing sites can be expected to be correlated with the presence of their corresponding nuclear specificity factors. The ever increasing amount of genome and transcriptome data offer a cornucopia of data to study this co-evolution of the nuclear and organelle genomes in the plant cell. We here present two cases of known editing factors, CRR28 and RARE1, affecting editing sites in *Arabidopsis thaliana* that we now found to be shared with *Amborella trichopoda*, hence spanning an evolutionary separation of more than 100 million years between the two extant flowering plants. RARE1 and CRR28 show highly different patterns of evolution among angiosperms that seem to correspond excellently to their known functionality.

RARE1 was found to perfectly co-exist with a requirement for its only known cognate RNA editing site accDeU794SL in the chloroplast *accD* gene in our sampling of 65 angiosperms. Numerous coinciding losses of the *accD* editing event and RARE1 have occurred independently during flowering plant evolution and suggest a surprisingly quick loss of RARE1 once it is obsolete owing to conversion of the cytidine to be edited into a thymidine or the loss of the *accD* gene from the chloroplast genome altogether. With this likely order of events it will be interesting to investigate further taxa branching phylogenetically close to the now identified loss events. We would predict to identify species representing “intermediate” cases of evolution where the *accD* RNA editing site has already been lost while RARE1 is still present, either in a functional or in an only mildly pseudogenized form. Such evolutionary scenarios have already been found for CRR4 and CRR21 on lower taxonomic levels (among Brassicaceae) once their cognate editing sites have vanished [46].

Interestingly, the accDeU794SL editing event affected by RARE1 is also present in the fern editomes (*Adiantum* and *Ophioglossum*) and in *Cycas taitungensis* [47] and we also predict it to exist in other gymnosperms like *Cathaya argyrophylla* and *Pinus thunbergii*. Hence, the phylogenetic history of RARE1 may reach deep into the vascular plant lineage. A complicating issue is that the unrelated editing factor VAC1 [48], also named ECB2 [49, 50], has also been shown to affect accDeU794SL editing in *Arabidopsis*. ECB2/VAC1, however additionally also targets editing site ndhFeU290SL, another editing event now found to be shared with *Amborella* (Fig. 3). Our preliminary analyses indeed suggest ECB2/VAC1 orthologues to also trace back at least into the angiosperm stem lineage (unpubl. obs.). Surprisingly, although editing event ndhFeU290SL is lost in addition to the loss of accDeU794SL in *Morus notabilis*, in *Vaccinium macrocarpon* and in the Poales, the ECB2/VAC1 orthologue appears to be retained in a functional form. We assume this to either indicate hitherto unidentified further transcript targets of ECB2/VAC1 or adaptations for new functionalities in the course of angiosperm evolution.

In contrast to RARE1, we only found evidence for one single loss of CRR28. We believe this finding to have its reason in CRR28 serving two important editing events simultaneously, ndhBeU467PL and ndhDeU878SL. As long as one of the two editing sites remains present, CRR28 likewise remains present, as we could observe for six cases of losing either the one or the other editing site in our sampling (Fig. 4). Only the loss of both chloroplast editing sites simultaneously can make CRR28 obsolete as here documented exclusively with the case of chickpea. Possibly, a taxon in the Fabales “bean” clade could be identified in the future where subsequent to the early loss of ndhBeU467PL and the later loss of ndhDeU878SL, the CRR28 gene is still retained.

A first report that correlated the absence of an editing site and a cognate protein factor biochemically was the case of psbEeU214PS and a 56 kDa protein, both of which are present in *Nicotiana* but absent in a pea *in vitro* RNA editing system [51]. The CREF3 locus has recently been identified as a specificity factor for the psbEeU214PS editing site in *Arabidopsis* [52], which is neither shared with *Amborella* nor *Cucumis* (Fig. 3), but with tobacco. A study based on a small, but phylogenetically wide taxon sampling of angiosperms already found the loss of the chloroplast *ndhD* start codon editing in *Manihot* and in Poaceae resulting from a genomic C-to-T conversion in the cpDNA to correlate well with the absence of detectable *crr4* gene orthologues [53]. Likewise, the recently identified mitochondrial RNA editing factor SMK1 appears to be lost quickly from plant genomes once the necessity of editing at its cognate site nad7eU836PL is lost [54]. Where an editing activity is maintained despite absence of a corresponding editing site this may either be due to additional functions, e.g. to target other editing sites in parallel like in the CRR28 case, or the only very recent loss of the editing site among closely related taxa [55].

While loss of organelle RNA editing sites and co-factors may altogether be dominating along the course of angiosperm evolution, there will also be several cases of novel editing sites appearing. Such new editing sites need to be served by new or modified nuclear editing factors, with or without gene duplication of already existing PPR genes. One obvious scenario for neo-functionalization is a change in the RNA-recognizing positions within the PPR arrays to allow binding to additional targets in the organelle transcriptomes. Investigating such scenarios will be extremely valuable to improve the present concept for the PPR-RNA binding code [43, 44, 56, 57]. Correctly assigning the PLS-type PPR repeats, extracting the amino acid positions relevant for RNA binding and properly translating them into likely RNA target sequences is currently very cumbersome and demands new bioinformatic approaches. Studying the evolution of RNA editing sites and their cognate co-factors and such novel bioinformatic approaches will clearly be mutually beneficial for an enhanced understanding of PPR-RNA-binding in the future. For that purpose, however, characterized RNA editing factors other than CRR28 or RARE1 with longer PPR arrays, showing more canonical RNA-binding according to the proposed code and ideally not affected by additional interacting proteins like the MORF/RIP proteins [58, 59] should best be investigated first. Along that lines, bryophytes and lycophytes may turn out to be an attractive option in the future, once more comparative genomic data become available, given the apparent absence of MORF/RIP proteins in those clades.

The study of RNA editing differences in nature or in chemically induced mutants has already proven useful to identify editing factors in forward genetic screens [39, 56, 60–64]. Conversely, the study of natural or mutated alleles or of different editing factor orthologues helps to reveal the underlying causes for different editing efficiencies at their cognate editing sites ([49], e.g. [65–68]).

In some cases, RNA editing sites and their nuclear co-factors may have co-evolved for much longer than only the age of angiosperms. It will be exciting to see whether RNA editing sites like the ones in *Amborella* that we find conserved in ferns, a lycophyte and even in a hornwort (Additional file 1) are acted upon by the same orthologous RNA editing factors co-existing since possibly 400 million years of plant evolution.

## Conclusion

Nuclear PPR protein genes encoding organelle RNA editing factors appear to disintegrate quickly after loss of their targets in plant chloroplasts. Comparing the here reported chloroplast editome of the early-branching *Amborella trichopoda* to plant molecular model organisms suggests coinciding losses of editing sites and their specificity factors to overall dominate in the course of angiosperm evolution. An editing factor like RARE1 addressing only a single editing target gets lost multiple times independently whereas loss of CRR28 can only occur once both its target cytidines are simultaneously converted into thymidines in a chloroplast genome. Contrary to the overall loss of RNA editing, many novel sites are also gained during flowering plant diversification. Exploring the evolutionary origin of their specificity factors will be an exciting future endeavour helping to improve our understanding of the RNA-PPR binding code. Ever more genomic sequences will offer vast data sets to study this molecular co-evolution across different genetic systems but will need to rely on novel bioinformatic tools and careful inspection and analyses of available data.

## Methods

### RNA editing prediction

Predictions of RNA editing for basal angiosperm chloroplast genome accessions of *Amborella trichopoda* (NC_005086), *Calycanthus floridus* (NC_004993), *Ceratophyllum demersum* (NC_009962), *Chloranthus spicatus* (NC_009598), *Cucumis sativus* (NC_007144), *Illicium oligandrum* (NC_009600), *Liriodendron tulipifera* (NC_008326), *Magnolia kwangsiensis* (NC_015892), *Nuphar advena* (NC_008788) and *Zingiber spectabile* (NC_020363) were done using PREPACT [22, 23] under http://www.prepact.de. Entire plastomes were used as input for the BLASTX mode of PREPACT to simultaneously identify protein coding genes and potential RNA editing events predicted from comparison to the 17 chloroplast editome references implemented in PREPACT 2.0. Thresholds were set to predictions from minimally 8 and at least 70 % of the references in the commons output (see Additional file 1). The assignment of RNA editing site labels according to the previously suggested nomenclature [16] was done using the cDNA mode of PREPACT.

### Plant material and molecular work

*Amborella trichopoda*, *Calycanthus floridus, Ceratophyllum demersum*, *Chloranthus spicatus*, *Illicium oligandrum*, *Liriodendron tulipifera, Magnolia kwangsiensis* and *Nuphar advena* material was obtained from the Botanical Garden Bonn. Cucumber (*Cucumis sativus*) and ginger (*Zingiber spectabile*) material was obtained commercially from a local grocery store. Total nucleic acid preparation was done using the CTAB method [69, 70]. RNA was alternatively isolated via the TRI Reagent Protocol (Sigma Aldrich). Synthesis of cDNA was accomplished using the Revert Aid First Strand cDNA Synthesis Kit (Thermo Scientific/Fermentas) in the presence of random hexamer primers or with gene-specific primers. Gene-specific primers were designed for PCR amplification of cDNAs. A complete list of oligonucleotides used for RT-PCR amplification is provided as Additional file 5. PCR products were recovered from agarose gels using the NucleoSpin Extract II Kit (Macherey & Nagel) and sequenced directly or after cloning into the pGEM-T Easy vector (Promega). Multiple cDNA clones were sequenced for each locus as indicated in Additional file 1, aiming for a coverage of ca. 5-fold (min. 3-fold) when verified edits immediately matched predictions, but increased to coverages of ca. 10-fold when predicted editing sites initially remained unidentified. Parallel inspection of the OneKP data (https://www.bioinfodata.org/Blast4OneKP/home) revealed that many editing events, including ones that we find efficiently edited here, are not represented in the assembled *Amborella* transcripts, likely owing to transcriptome assembly strategies. Conversely, four exceptional cases of editing identified there but not in our study are highlighted in Additional file 1.

### Sequence handling and phylogenetic analyses

Sequence handling and analyses was mainly done using the MEGA alignment feature [71]. *Arabidopsis thaliana* protein sequences of RNA editing factors CRR28 (NP_176180.1) and RARE1 (NP_196831.1) were used as queries to search for homologues in other angiosperm genomes both by standard protein BLASTP and by TBLASTN of whole genome shotgun or transcribed RNA sequences at the NCBI BLAST server available at http://blast.ncbi.nlm.nih.gov/Blast.cgi [72, 73]. Given the repetitive nature of the pentatricopeptide repeat (PPR) arrays, care was taken to include orthologues and avoid the inclusion of paralogues through repeated phylogenetic analyses. To this end, top BLAST hits were added into alignments that were iteratively used for construction and inspection of phylogenetic trees. Further addition of homologous sequences was stopped when obvious paralogue duplicates branching outside of the ingroup, as delimited by the respective “basal” *Amborella* orthologue, were encountered. For clarity, only the copy more similar to other orthologues was retained for species where duplicates likely originating from alternative gene models were present. The angiosperm protein sampling excluded more exotic taxa for which no plastome sequences have as yet been assembled (e.g. *Diospyros, Humulus, Leersia, Leavenworthia, Sisymbrium* and *Ziziphus*), taxa for which genome sequence assembly qualities are insufficient at present to reliably identify protein orthologues (e.g. *Genlisea, Lagenaria, Malus, Momordica, Rauvolfia* and *Rhazya*) and, for clarity, very closely related proteins from species of the same genus (e.g. *Agave, Arachis, Citrus, Eucalyptus, Eutrema, Gossypium, Hordeum, Oryza, Prunus* and *Triticum*). Two species each were included, however, for genera *Arabidopsis, Cucumis, Nicotiana* and *Solanum* that include more widely investigated RNA editing model organisms.

Protein sequence alignments and phylogenetic analyses were done using MEGA. Given the repetitive nature of the PPRs, careful manual alignment correction was needed subsequent to automatic alignment by CLUSTAL as implemented in MEGA. Phylogenetic tree construction of the CRR28 and RARE1 alignments was done with the Maximum Likelihood (ML) model using the JTT + Γ + I + F and the JTT + Γ + I model, respectively, which were selected as best-fitting models of sequence evolution. Only alignment positions with at least 90 % coverage were included resulting in 517 and 775 alignment positions included for CRR28 and RARE1 phylogenetic tree constructions, respectively. Node supports were determined with 100 bootstrapping replicates. A cladogram representing a wide selection of angiosperms for which high-quality genome data are available was created by manually editing a NEWICK file based on generally accepted insights of angiosperm phylogeny, as for example reflected in the recently realized “Open Tree of Life” project [74] section on Magnoliophyta (https://tree.opentreeoflife.org/opentree/opentree3.0@64078/Magnoliophyta and references therein). Phylogenetic trees were created and edited using the MEGA Tree Explorer feature.

## Additional files

Additional file 1: Table S1. RNA editing events identified in *Amborella trichopoda* chloroplast mRNAs sorted by gene names. (DOCX 41 kb) Additional file 2:
**Prediction of RNA editing using the BLASTX prediction mode of PREPACT exemplarily shown for the** ***Amborella trichopoda ndhD*** **gene.** (DOCX 66 kb) Additional file 3:
**Phylogenetic tree of angiosperm homologues to** ***Arabidopsis thaliana*** **CRR28.** (DOCX 22 kb) Additional file 4:
**Phylogenetic tree of angiosperm homologues to** ***Arabidopsis thaliana*** **RARE1.** (DOCX 19 kb) Additional file 5:
**Chloroplast primer sequences.** (DOCX 34 kb)

## References

[1] Covello PS, Gray MW (1989). RNA editing in plant mitochondria. Nature.

[2] Gualberto JM, Lamattina L, Bonnard G, Weil JH, Grienenberger JM (1989). RNA editing in wheat mitochondria results in the conservation of protein sequences. Nature.

[3] Hiesel R, Wissinger B, Schuster W, Brennicke A (1989). RNA editing in plant mitochondria. Science.

[4] Hoch B, Maier RM, Appel K, Igloi GL, Kössel H (1991). Editing of a chloroplast mRNA by creation of an initiation codon. Nature.

[5] Kotera E, Tasaka M, Shikanai T (2005). A pentatricopeptide repeat protein is essential for RNA editing in chloroplasts. Nature.

[6] Takenaka M, Verbitskiy D, Zehrmann A, Härtel B, Bayer-Császár E, Glass F (2014). RNA editing in plant mitochondria -- Connecting RNA target sequences and acting proteins. Mitochondrion.

[7] Fujii S, Small I (2011). The evolution of RNA editing and pentatricopeptide repeat genes. New Phytol.

[8] Schmitz-Linneweber C, Small I (2008). Pentatricopeptide repeat proteins: a socket set for organelle gene expression. Trends Plant Sci.

[9] Finster S, Legen J, Qu Y, Schmitz-Linneweber C, Bock R, Knoop V (2012). Land Plant RNA Editing or: Don’t Be Fooled by Plant Organellar DNA Sequences. Genomics of Chloroplasts and Mitochondria.

[10] Chateigner-Boutin ALA-L, Small I (2010). Plant RNA editing. RNA Biol.

[11] Barkan A, Small I (2014). Pentatricopeptide repeat proteins in plants. Annu Rev Plant Biol.

[12] Shikanai T (1874). RNA editing in plants: Machinery and flexibility of the site recognition. Biochim Biophys Acta Bioenerg.

[13] Knoop V: When you can’t trust the DNA: RNA editing changes transcript sequences. Cellular and Molecular Life Sciences 2011:567–586 10.1007/s00018-010-0538-9 PMC11114842 20938709

[14] Takenaka M, Zehrmann A, Verbitskiy D, Härtel B, Brennicke A (2013). RNA editing in plants and its evolution. Annu Rev Genet.

[15] Miyata Y, Sugita M (2004). Tissue- and stage-specific RNA editing of *rps14* transcripts in moss (*Physcomitrella patens*) chloroplasts. J Plant Physiol.

[16] Rüdinger M, Funk HT, Rensing SA, Maier UG, Knoop V (2009). RNA editing: only eleven sites are present in the *Physcomitrella patens* mitochondrial transcriptome and a universal nomenclature proposal. Mol Genet Genomics.

[17] Ichinose M, Sugita C, Yagi Y, Nakamura T, Sugita M (2013). Two DYW subclass PPR proteins are involved in RNA editing of ccmFc and atp9 transcripts in the moss Physcomitrella patens: first complete set of PPR editing factors in plant mitochondria. Plant Cell Physiol.

[18] Schallenberg-Rüdinger M, Kindgren P, Zehrmann A, Small I, Knoop V (2013). A DYW-protein knockout in *Physcomitrella* affects two closely spaced mitochondrial editing sites and causes a severe developmental phenotype. Plant J.

[19] Ichinose M, Uchida M, Sugita M (2014). Identification of a pentatricopeptide repeat RNA editing factor in *Physcomitrella patens* chloroplasts. FEBS Lett.

[20] Oldenkott B, Yamaguchi K, Tsuji-Tsukinoki S, Knie N, Knoop V (2014). Chloroplast RNA editing going extreme: more than 3400 events of C-to-U editing in the chloroplast transcriptome of the lycophyte *Selaginella uncinata*. RNA.

[21] Hecht J, Grewe F, Knoop V (2011). Extreme RNA editing in coding islands and abundant microsatellites in repeat sequences of *Selaginella moellendorffii* mitochondria: the root of frequent plant mtDNA recombination in early tracheophytes. Genome Biol Evol.

[22] Lenz H, Knoop V (2013). PREPACT 2.0: Predicting C-to-U and U-to-C RNA Editing in Organelle Genome Sequences with Multiple References and Curated RNA Editing Annotation. Bioinform Biol Insights.

[23] Lenz H, Rüdinger M, Volkmar U, Fischer S, Herres S, Grewe F (2010). Introducing the plant RNA editing prediction and analysis computer tool PREPACT and an update on RNA editing site nomenclature. Curr Genet.

[24] Shields DC, Wolfe KH (1997). Accelerated evolution of sites undergoing mRNA editing in plant mitochondria and chloroplasts. Mol Biol Evol.

[25] Mower JP (2008). Modeling sites of RNA editing as a fifth nucleotide state reveals progressive loss of edited sites from angiosperm mitochondria. Mol Biol Evol.

[26] Cuenca A, Petersen G, Seberg O, Davis JI, Stevenson DW (2010). Are substitution rates and RNA editing correlated?. BMC Evol Biol.

[27] Sloan DB, MacQueen AH, Alverson AJ, Palmer JD, Taylor DR (2010). Extensive loss of RNA editing sites in rapidly evolving silene mitochondrial genomes: selection vs. retroprocessing as the driving force. Genetics.

[28] Richardson AO, Rice DW, Young GJ, Alverson AJ, Palmer JD (2013). The “fossilized” mitochondrial genome of *Liriodendron tulipifera*: ancestral gene content and order, ancestral editing sites, and extraordinarily low mutation rate. BMC Biol.

[29] Wolf PG, Rowe CA, Hasebe M (2004). High levels of RNA editing in a vascular plant chloroplast genome: analysis of transcripts from the fern Adiantum capillus-veneris. Gene.

[30] Guo W, Grewe F, Mower JP (2015). Variable frequency of plastid RNA editing among ferns and repeated loss of uridine-to-cytidine editing from vascular plants. PLoS One.

[31] Kugita M, Yamamoto Y, Fujikawa T, Matsumoto T, Yoshinaga K (2003). RNA editing in hornwort chloroplasts makes more than half the genes functional. Nucleic Acids Res.

[32] Guzowska-Nowowiejska M, Fiedorowicz E, Plader W (2009). Cucumber, melon, pumpkin, and squash: Are rules of editing in flowering plants chloroplast genes so well known indeed?. Gene.

[33] Lutz KAA, Maliga P (2001). Lack of conservation of editing sites in mRNAs that encode subunits of the NAD(P)H dehydrogenase complex in plastids and mitochondria of *Arabidopsis thaliana*. Curr Genet.

[34] Tillich M, Funk HT, Schmitz-Linneweber C, Poltnigg P, Sabater B, Martin M (2005). Editing of plastid RNA in *Arabidopsis thaliana* ecotypes. Plant J.

[35] Ruwe H, Castandet B, Schmitz-Linneweber C, Stern DB (2013). *Arabidopsis* chloroplast quantitative editotype. FEBS Lett.

[36] Fiebig A, Stegemann S, Bock R (2004). Rapid evolution of RNA editing sites in a small non-essential plastid gene. Nucleic Acids Res.

[37] Okuda K, Chateigner-Boutin ALA-L, Nakamura T, Delannoy E, Sugita M, Myouga F (2009). Pentatricopeptide repeat proteins with the DYW motif have distinct molecular functions in RNA Editing and RNA cleavage in Arabidopsis chloroplasts. Plant Cell.

[38] Sun T, Shi X, Friso G, Van Wijk K, Bentolila S, Hanson MR (2015). A zinc finger motif-containing protein is essential for chloroplast RNA editing. PLoS Genet.

[39] Robbins JC, Heller WP, Hanson MR (2009). A comparative genomics approach identifies a PPR-DYW protein that is essential for C-to-U editing of the *Arabidopsis* chloroplast *accD* transcript. RNA.

[40] Boussardon C, Avon A, Kindgren P, Bond CSCS, Challenor M, Lurin C (2014). The cytidine deaminase signature HxE(x)nCxxC of DYW1 binds zinc and is necessary for RNA editing of ndhD-1. New Phytol.

[41] Salone V, Rüdinger M, Polsakiewicz M, Hoffmann B, Groth-Malonek M, Szurek B (2007). A hypothesis on the identification of the editing enzyme in plant organelles. FEBS Lett.

[42] Hayes ML, Dang KN, Diaz MF, Mulligan RM (2015). A Conserved Glutamate Residue in the C-terminal Deaminase Domain of Pentatricopeptide Repeat Proteins is Required for RNA Editing Activity. J Biol Chem.

[43] Barkan A, Rojas M, Fujii S, Yap A, Chong YS, Bond CS (2012). A combinatorial amino acid code for RNA recognition by pentatricopeptide repeat proteins. PLoS Genet.

[44] Yagi Y, Hayashi S, Kobayashi K, Hirayama T, Nakamura T (2013). Elucidation of the RNA recognition code for pentatricopeptide repeat proteins involved in organelle RNA editing in plants. PLoS One.

[45] Tillich M, Lehwark P, Morton BR, Maier UG (2006). The evolution of chloroplast RNA editing. Mol Biol Evol.

[46] Hayes ML, Giang K, Mulligan RM (2012). Molecular evolution of pentatricopeptide repeat genes reveals truncation in species lacking an editing target and structural domains under distinct selective pressures. BMC Evol Biol.

[47] Chen H, Deng L, Jiang Y, Lu P, Yu J (2011). RNA editing sites exist in protein-coding genes in the chloroplast genome of *Cycas taitungensis*. J Integr Plant Biol.

[48] Tseng C-C, Sung T-Y, Li Y-C, Hsu S-J, Lin C-L, Hsieh M-H (2010). Editing of *accD* and *ndhF* chloroplast transcripts is partially affected in the *Arabidopsis* vanilla cream1 mutant. Plant Mol Biol.

[49] Cao ZL, Yu QB, Sun Y, Lu Y, Cui YL, Yang ZN (2011). A point mutation in the pentatricopeptide repeat motif of the AtECB2 protein causes delayed chloroplast development. J IntegrPlant Biol.

[50] Yu QBQ-B, Jiang Y, Chong K, Yang ZNZ-N (2009). AtECB2, a pentatricopeptide repeat protein, is required for chloroplast transcript accD RNA editing and early chloroplast biogenesis in *Arabidopsis thaliana*. Plant J.

[51] Miyamoto T, Obokata J, Sugiura M (2002). Recognition of RNA editing sites is directed by unique proteins in chloroplasts: biochemical identification of cis-acting elements and trans-acting factors involved in RNA editing in tobacco and pea chloroplasts. Mol Cell Biol.

[52] Yagi Y, Tachikawa M, Noguchi H, Satoh S, Obokata J, Nakamura T (2013). Pentatricopeptide repeat proteins involved in plant organellar RNA editing. RNA Biol.

[53] Hayes ML, Mulligan RM (2011). Pentatricopeptide repeat proteins constrain genome evolution in chloroplasts. Mol Biol Evol.

[54] Li X-J, Zhang Y-F, Hou M, Sun F, Shen Y, Xiu Z-H (2014). Small kernel 1 encodes a pentatricopeptide repeat protein required for mitochondrial *nad7* transcript editing and seed development in maize (*Zea mays*) and rice (*Oryza sativa*). Plant J.

[55] Tillich M, Poltnigg P, Kushnir S, Schmitz-Linneweber C (2006). Maintenance of plastid RNA editing activities independently of their target sites. EMBO Rep.

[56] Nakamura T, Yagi Y, Kobayashi K (2012). Mechanistic insight into pentatricopeptide repeat proteins as sequence-specific RNA-binding proteins for organellar RNAs in plants. Plant Cell Physiol.

[57] Takenaka M, Zehrmann A, Brennicke A, Graichen K (2013). Improved computational target site prediction for pentatricopeptide repeat RNA editing factors. PLoS One.

[58] Takenaka M, Zehrmann A, Verbitskiy D, Kugelmann M, Härtel B, Brennicke A (2012). Multiple organellar RNA editing factor (MORF) family proteins are required for RNA editing in mitochondria and plastids of plants. Proc Natl Acad Sci.

[59] Bentolila S, Heller WP, Sun T, Babina AM, Friso G, van Wijk KJ (2012). RIP1, a member of an Arabidopsis protein family, interacts with the protein RARE1 and broadly affects RNA editing. Proc Natl Acad Sci U S A.

[60] Bentolila S, Knight W, Hanson M (2010). Natural variation in Arabidopsis leads to the identification of REME1, a pentatricopeptide repeat-DYW protein controlling the editing of mitochondrial transcripts. Plant Physiol.

[61] Bentolila S, Babina AM, Germain A, Hanson MR (2013). Quantitative trait locus mapping identifies REME2, a PPR-DYW protein required for editing of specific C targets in *Arabidopsis* mitochondria. RNA Biol.

[62] Zehrmann A, Verbitskiy D, van der Merwe JA, Brennicke A, Takenaka M (2009). A DYW domain-containing pentatricopeptide repeat protein is required for RNA editing at multiple sites in mitochondria of Arabidopsis thaliana. Plant Cell.

[63] Zehrmann A, van der Merwe JA, Verbitskiy D, Härtel B, Brennicke A, Takenaka M (2012). The DYW-class PPR protein MEF7 is required for RNA editing at four sites in mitochondria of *Arabidopsis thaliana*. RNA Biol.

[64] Härtel B, Zehrmann A, Verbitskiy D, van der Merwe JA, Brennicke A, Takenaka M (2013). MEF10 is required for RNA editing at *nad2*-842 in mitochondria of *Arabidopsis thaliana* and interacts with MORF8. Plant Mol Biol.

[65] Okuda K, Habata Y, Kobayashi Y, Shikanai T (2008). Amino acid sequence variations in Nicotiana CRR4 orthologs determine the species-specific efficiency of RNA editing in plastids. Nucleic Acids Res.

[66] Glass F, Härtel B, Zehrmann A, Verbitskiy D, Takenaka M: MEF13 requires MORF3 and MORF8 for RNA editing at eight targets in mitochondrial mRNAs in Arabidopsis thaliana. Mol Plant 2015. 10.1016/j.molp.2015.05.008 26048647

[67] Zehrmann A, Verbitskiy D, Härtel B, Brennicke A, Takenaka M (2010). RNA editing competence of trans-factor MEF1 is modulated by ecotype-specific differences but requires the DYW domain. FEBS Lett.

[68] Verbitskiy D, Zehrmann A, Härtel B, Brennicke A, Takenaka M (2012). Two related RNA-editing proteins target the same sites in mitochondria of *Arabidopsis thaliana*. J Biol Chem.

[69] Doyle JLJJ, Doyle JLJJ (1990). Isolation of plant DNA from fresh tissue. Focus (Madison).

[70] Liao Z, Chen M, Guo L, Gong Y, Tang F, Sun X (2004). Rapid isolation of high-quality total RNA from taxus and ginkgo. Prep Biochem Biotechnol.

[71] Tamura K, Stecher G, Peterson D, Filipski A, Kumar S (2013). MEGA6: Molecular Evolutionary Genetics Analysis version 6.0.. Mol Biol Evol.

[72] Altschul SF, Madden TL, Schaffer AA, Zhang J, Zhang Z, Miller W (1997). Gapped BLAST and PSI-BLAST: a new generation of protein database search programs. Nucleic Acids Res.

[73] Altschul SF, Gish W, Miller W, Myers EW, Lipman DJ (1990). Basic local alignment search tool. J Mol Biol.

[74] Hinchliff CE, Smith SA, Allman JF, Burleigh JG, Chaudhary R, Coghill LM (2015). Synthesis of phylogeny and taxonomy into a comprehensive tree of life. Proc Natl Acad Sci.

[75] Lohse M, Drechsel O, Bock R (2007). Organellar Genome DRAW (OGDRAW): a tool for the easy generation of high-quality custom graphical maps of plastid and mitochondrial genomes. Curr Genet.

[76] Crooks GE, Hon G, Chandonia JM, Brenner SE (2004). WebLogo: a sequence logo generator. Genome Res.

