# Supplementary material for: Frequent chloroplast RNA editing in early-branching flowering plants: pilot studies on angiosperm-wide coexistence of editing sites and their nuclear specificity factors
Source: BMC Evol Biol. 2016 Jan 25;16:23. doi: 10.1186/s12862-016-0589-0 (PMC4727281; doi:10.1186/s12862-016-0589-0)
Supplement: Additional file 1: Table S1. — RNA editing events identified in Amborella trichopoda chloroplast mRNAs sorted by gene names. (DOCX 41 kb) [file 12862_2016_589_MOESM1_ESM.docx]

**Supplementary Table 1.** RNA editing events identified in *Amborella trichopoda* chloroplast mRNAs sorted by gene names. RNA editing is exclusively of the C-to-U type (‘eU’). Editing site nomenclature is as proposed previously [16], indicating the position in the reading frame and the amino acid codon identity before and after editing, respectively. The letter ‘P’ behind the respective gene name indicates perfect agreement between cDNA analysis and PREPACT prediction (from at least 70% and minimally 8 hits among the 17 references implemented in PREPACT 2) for a given gene. Additional sites of RNA editing below the prediction threshold or unpredictable silent sites are underlined and, *vice versa*, predicted sites of editing remaining unconfirmed are given in grey italics and are commented. The number of edited vs. total cDNAs covering a site is indicated in the fourth column (‘cov.’). Numerous sites including some found strongly edited here are not represented by the *Amborella* OneKP transcriptome assembly. *Vice versa*, four exceptional cases of expected edits identified there but not in our study are indicated by asterisks (ndhGeU133RC, psbKeU173AV, rpl16eU310HY and ycf2eU6761PL). The letter ‘N’ indicates novel sites of chloroplast RNA editing not observed previously or (in parentheses) only outside of angiosperms as also commented.

| **Gene** |  | **edits** | **cov.** |  | **comments** |
| --- | --- | --- | --- | --- | --- |
| ***accD*** |  | eU923SL | 10/10 |  |  |
|  |  | eU1122FF | 3/10 |  |  |
|  |  | eU1286SL | 9/10 |  |  |
|  |  | *eU1490SL* | 1/10 |  | Unexplained lack of editing. Edit in *Ophioglossum*. |
|  |  | eU1514SL | 4/10 |  |  |
|  |  | eU1532PL | 10/10 |  |  |
|  |  | eU1547PL | 8/10 | (N) | *Anthoceros* edit. |
| ***atpA*** | P | eU194PL | 5/5 | (N) | *Adiantum* edit. |
|  |  | eU781HY | 5/5 | N |  |
|  |  | eU791PL | 5/5 |  |  |
|  |  | eU914SL | 5/5 |  |  |
|  |  | eU1148SL | 5/5 |  |  |
| ***atpB*** | P | eU833SF | 5/5 | (N) | *Selaginella* edit. |
|  |  | eU1235PL | 5/5 | (N) | *Anthoceros* edit. |
| ***atpF*** | P | eU92PL | 5/5 |  |  |
| ***atpI*** | P | eU352RW | 5/5 | (N) | *Selaginella* edit. |
|  |  | eU431PL | 5/5 |  |  |
|  |  | eU632SL | 5/5 |  |  |
| ***ccsA*** |  | eU68TM | 9/10 | N | 0% prediction. |
|  |  | *eU71TI* | 0/10 |  | No conservation of I outside of angiosperms. |
|  |  | eU148RC | 8/10 | N | 67% prediction. |
|  |  | *eU626TI* | 0/10 |  | Unedited also in *Cocos, Gossypium, Zea*. No conservation of I outside of angiosperms. |
|  |  | eU785SF | 6/10 |  |  |
| ***cemA*** |  | *eU139LF* | 0/10 |  | Unedited also in *Oryza* and *Phalaenopsis*. L also retained in *Chara*. |
|  |  | *eU323SL* | 0/10 |  | Unedited also in *Ophioglossum*. |
|  |  | *eU398TI* | 0/10 |  | Unexplained lack of editing. Occasionally V outside of angiosperms. |
|  |  | eU479SL | 10/10 | (N) | *Ophioglossum* edit. |
| ***clpP*** | P | eU82HY | 5/5 |  |  |
|  |  | eU559HY | 5/5 |  |  |
| ***ndhA*** |  | eU107PL | 8/12 | N |  |
|  |  | eU113SL | 9/12 | (N) | *Selaginella* edit. |
|  |  | *eU337HY* | 0/12 |  | Unedited also in *Selaginella*. Occasionally F elsewhere. |
|  |  | eU395SF | 12/12 | N |  |
|  |  | eU476SL | 11/12 |  |  |
|  |  | eU566SL | 12/12 |  |  |
|  |  | eU961PS | 11/11 |  |  |
|  |  | eU1073SF | 6/6 |  |  |
| ***ndhB*** | P | eU93FF | 6/6 |  | silent. |
|  |  | eU149SL | 6/6 |  |  |
|  |  | eU467PL | 3/8 |  |  |
|  |  | eU542TM | 8/8 |  |  |
|  |  | eU586HY | 7/8 |  |  |
|  |  | eU611SL | 6/8 |  |  |
|  |  | eU704SF | 8/8 |  |  |
|  |  | eU737PL | 7/8 |  |  |
|  |  | eU830SL | 8/9 |  |  |
|  |  | eU1102RC | 8/9 |  |  |
|  |  | eU1112SL | 6/9 |  |  |
|  |  | eU1121PL | 6/8 |  |  |
|  |  | eU1193SL | 6/8 | (N) | *Anthoceros* and *Adiantum* edit. |
|  |  | eU1255HY | 6/8 |  |  |
|  |  | eU1481PL | 6/7 |  |  |
| ***ndhC*** | P | eU311PL | 5/5 | (N) | *Selaginella* edit. |
|  |  | eU323SL | 5/5 |  |  |
| ***ndhD*** | P | eU2TM | 6/10 |  |  |
|  |  | eU59SL | 10/10 | (N) | *Anthoceros, Ophioglossum* edit. |
|  |  | eU305SL | 6/10 | (N) | *Anthoceros* edit. |
|  |  | eU383SL | 6/10 |  |  |
|  |  | eU599SL | 6/10 |  |  |
|  |  | eU668AV | 10/10 | (N) |  |
|  |  | eU674SL | 6/10 |  |  |
|  |  | eU688LL | 6/10 |  | silent. |
|  |  | eU878SL | 4/10 |  |  |
|  |  | eU944SF | 3/10 | (N) | *Selaginella* edit. |
|  |  | eU947TI | 4/10 |  | 50% prediction. |
|  |  | eU1163SL | 4/10 | (N) | *Selaginella* edit. |
|  |  | eU1310SL | 6/10 |  |  |
| ***ndhE*** | P | eU233PL | 10/10 |  |  |
| ***ndhF*** |  | eU242SF | 5/5 | N | 63% prediction. |
|  |  | eU290SL | 5/5 |  |  |
|  |  | eU482SL | 5/5 | (N) | *Selaginella* edit. |
|  |  | eU586LF | 5/5 | N | 69% prediction. |
|  |  | *eU644SL* | 0/5 |  | W in Poales, T in *Chara*. |
|  |  | eU1010SL | 5/5 | (N) | *Anthoceros* edit. |
|  |  | eU1046SL | 5/5 | N |  |
| ***ndhG*** |  | eU116SL | 9/10 | (N) | *Adiantum* and *Selaginella* edit. |
|  |  | *eU133RC* | 0/10* |  | Edit in *Pellia*, but occasionally S or L elsewhere. |
|  |  | eU148HY | 4/10 | (N) | *Selaginella* edit. |
| **ndhH** |  | eU505HY | 10/11 |  |  |
|  |  | *eU1075LF* | 0/11 |  | Unexplained lack of editing. |
| **ndhI** |  | *eU140SL* | 0/10 |  | Unedited also in monocots. |
|  |  | *eU442PS* | 0/10 |  | Unedited also in Monilophytes. |
| ***ndhJ*** | P | eU128SL | 5/5 | (N) | *Adiantum* and *Selaginella* edit. |
| ***ndhK*** |  | eU65PL | 5/5 |  | 50% prediction. |
|  |  | *eU631LF* | 0/5 |  |  |
| ***petB*** | P | eU100HY | 2/3 |  |  |
|  |  | eU418RW | 3/3 |  |  |
|  |  | eU611PL | 3/3 |  |  |
| ***petG*** | P | eU17PL | 13/13 | N |  |
| ***petL*** |  | eU5PL | 2/13 |  |  |
|  |  | eU23SF | 12/13 | N |  |
|  |  | eU44SL | 11/13 | N |  |
| ***psaI*** | P | eU85HY | 3/5 |  |  |
| ***psbJ*** | P | eU59PL | 5/5 |  |  |
|  |  | eU71SL | 5/5 | (N) | *Selaginella* edit. |
| ***psbK*** |  | *eU5PL* | 0/5 |  | Unedited also in Poales. |
|  |  | eU15FF | 4/5 |  | silent. |
|  |  | eU173AV | 0/5* |  | 96 % prediction. |
| ***psbL*** | P | eU2TM | 5/5 |  |  |
|  |  | eU92SL | 5/5 | (N) | *Adiantum* and *Selaginella* edit. |
| ***psbN*** |  | *eU41SL* | 0/10 |  | Unedited also in *Adiantum, Equisetum* and *Physcomitrella.* |
| ***rpl2*** | P | eU2TM | 5/5 |  |  |
| ***rpl16*** |  | *eU310HY* | 0/10* |  | Unexplained lack of editing. Edit in *Anthoceros, Ophioglossum* and *Selaginella.* |
| ***rpl20*** | P | eU284PL | 5/5 | N |  |
| ***rpl22*** | P | eU227SL | 3/3 |  |  |
| ***rpl23*** | P | eU71SF | 5/5 |  |  |
| ***rpoA*** |  | *eU140TI* | 0/10 |  | Unedited also in *Pellia.* |
|  |  | eU200SF | 10/10 |  | 50% prediction, edited in some but not in other angiosperms. |
|  |  | eU368SL | 10/10 |  |  |
|  |  | eU521SF | 10/10 | (N) | *Anthoceros* and *Selaginella* edit. |
| ***rpoB*** |  | eU29SF | 4/10 |  | 23% prediction. |
|  |  | *eU157LF* | 0/10 |  | Unedited also in *Ophioglossum* and *Physcomitrella*. Not conserved in algae. |
|  |  | *eU226HY* | 0/10 |  | Unexplained lack of editing. |
|  |  | eU460RW | 7/10 | N |  |
|  |  | eU473SL | 4/10 |  |  |
|  |  | eU551PL | 6/10 |  |  |
|  |  | eU566SL | 4/10 |  |  |
|  |  | *eU623PL* | 0/10 |  | Unedited also in *Oryza.* |
|  |  | eU1178SL | 5/10 | (N) | *Selaginella* edit. |
|  |  | eU1981HY | 7/10 |  |  |
|  |  | eU2432SL | 10/10 |  |  |
|  |  | eU2710PS | 9/10 | (N) | *Selaginella* edit. |
|  |  | eU2926LF | 5/10 |  | 0% prediction. |
| ***rpoC1*** |  | eU222YY | 3/5 |  | silent. |
|  |  | eU281SF | 3/5 | (N) | *Ophioglossum* edit. |
|  |  | *eU389SL* | 0/10 |  | Unexplained lack of editing. Edit in *Selaginella*. |
|  |  | eU488SL | 4/10 |  |  |
|  |  | eU511RW | 5/10 |  |  |
|  |  | eU552FF | 10/10 |  | silent. |
|  |  | *eU617PL* | 0/10 |  | Unexplained lack of editing. Edit in *Selaginella, Phalaenopsis, Cocos*. |
|  |  | eU635TI | 10/10 |  | 65% prediction. |
|  |  | *eU760LF* | 0/10 |  | Unexplained lack of editing. Edit in *Selaginella*. |
|  |  | eU787RW | 5/5 | N |  |
|  |  | eU860PL | 5/5 | N |  |
|  |  | eU926PL | 5/5 |  | 65% prediction. |
|  |  | eU959SL | 4/5 | N |  |
|  |  | eU1447RC | 5/5 | (N) | *Adiantum* and *Selaginella* edit. |
|  |  | eU1912HY | 4/5 | (N) | *Selaginella* edit. |
| ***rpoC2*** |  | eU536TI | 3/7 | N |  |
|  |  | eU2303SL | 4/10 |  |  |
|  |  | eU2819SF | 9/10 |  | 35% prediction. |
|  |  | *eU3014SL* | 0/10 |  | Weak conservation, e.g. F in *Cocos* and *Phalaenopsis*. |
|  |  | *eU3286HY* | 0/10 |  | S in *Anthoceros*, F in *Adiantum*. |
|  |  | *eU3635TI* | 0/10 |  | M in *Selaginella*. |
|  |  | eU3698SL | 10/10 |  |  |
| ***rps2*** | P | eU134TI | 5/5 |  | 65% prediction. |
|  |  | eUU158PL | 5/5 | N | (conserved as L or F) |
|  |  | eU248SL | 5/5 |  |  |
|  |  | eU314SL | 5/5 | (N) | *Anthoceros* and *Selaginella* edit. |
| ***rps3*** | P | eU239SF | 3/3 |  |  |
| ***rps8*** | P | eU293SL | 4/5 | (N) | *Selaginella* edit. |
| ***rps12*** | P | eU221SL | 5/5 |  |  |
| ***rps14*** |  | eU149PL | 5/5 |  |  |
|  |  | *eU194SL* | 0/5 |  | Unedited also in *Arabidopsis, Gossypium* and *Hevea.* |
| ***rps16*** | P | eU143SL | 5/5 |  |  |
|  |  | eU212SL | 5/5 | (N) | *Anthoceros* edit. |
| ***rps18*** | P | eU221PL | 5/5 |  |  |
| ***ycf1*** |  | *eU2089LF* | 0/5 |  | Unedited also in *Adiantum, Anthoceros, Equisetum, Ophioglossum, Physcomitrella,* and *Selaginella*. |
|  |  | *eU3620SL* | 0/10 |  | Unedited also in *Cocos* and *Ophioglossum*. |
|  |  | eU5195SL | 8/10 | N |  |
|  |  | *eU5242PS* | 0/10 |  | Unedited also in *Ophioglossum*, L in *Arabidopsis, Physcomitrella* and *Psilotum.* |
| ***ycf2*** |  | *eU119TI* | 0/10 |  | Unedited also in *Anthoceros, Ophioglossum* and *Cocos*. |
|  |  | *eU3170SL* | 0/10 |  | S in *Psilotum*, F in *Physcomitrella*. |
|  |  | *eU3745LF* | 0/10 |  | Unedited also in *Cocos, Phalaenopsis* and *Marchantia*, P in *Anthoceros*. |
|  |  | *eU4250PL* | 0/10 |  | S in *Physcomitrella*. |
|  |  | *eU5216PL* | 0/10 |  | S in *Chara*. |
|  |  | eU5432SL | 9/10 | (N) | *Selaginella* edit. |
|  |  | *eU5891TI* | 0/5 |  | R in *Selaginella*, M in *Adiantum*, L in *Anthoceros, Equisetum* and *Psilotum*. |
|  |  | *eU6761PL* | 0/5* |  | Unedited also in *Gossypium*, R in *Arabidopsis*. |
| ***ycf3*** | P | eU44SF | 5/5 |  |  |
|  |  | eU185TM | 5/5 |  |  |
|  |  | eU191PL | 5/5 |  |  |
|  |  | eU326SF | 5/5 | N |  |
|  |  | eU407SF | 5/5 | (N) | *Selaginella* edit. |
| **ycf4** | P | eU553R* | 3/5 |  |  |
