# Supplementary material for: Frequent chloroplast RNA editing in early-branching flowering plants: pilot studies on angiosperm-wide coexistence of editing sites and their nuclear specificity factors
Source: BMC Evol Biol. 2016 Jan 25;16:23. doi: 10.1186/s12862-016-0589-0 (PMC4727281; doi:10.1186/s12862-016-0589-0)
Supplement: Additional file 2: — Prediction of RNA editing using the BLASTX prediction mode of PREPACT exemplarily shown for the Amborella trichopoda ndhD gene. (DOCX 66 kb) [file 12862_2016_589_MOESM2_ESM.docx]

**Additional File 2**


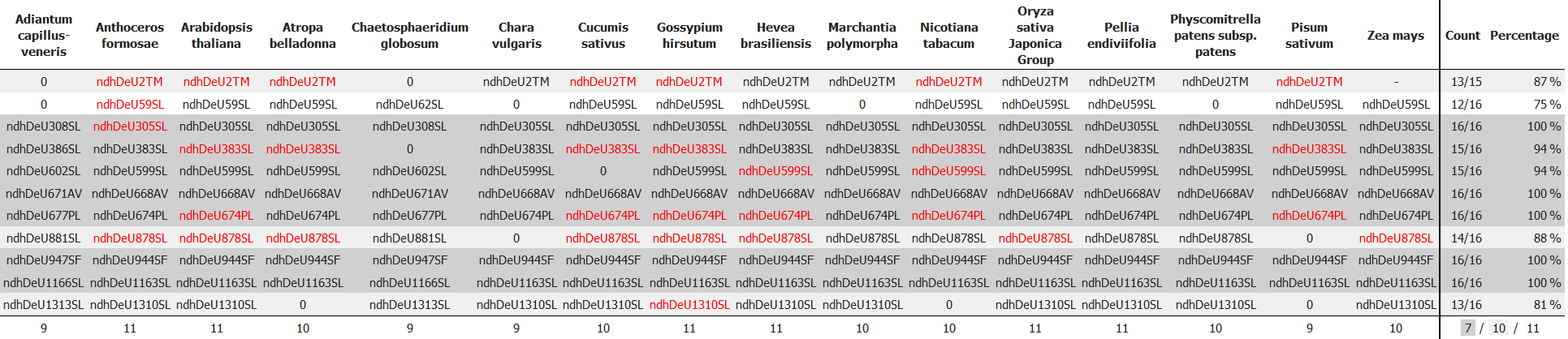
 ...

...
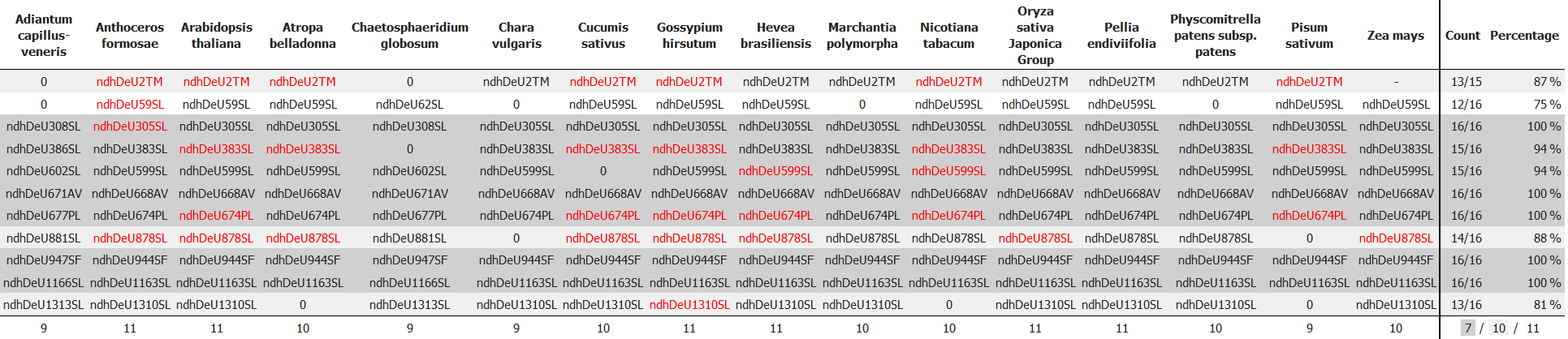


**Additional File 2**. **Prediction of RNA editing** **using the BLASTX prediction mode of PREPACT** exemplarily shown for the *Amborella trichopoda* *ndhD* gene. All 17 chloroplast references implemented in PREPACT 2 [22] were selected as references to predict RNA editing using a threshold for listing sites congruently predicted by at least 70% (and minimally 8) of the references. Light and dark grey shading additionally indicates threshold confidence levels of 80% and 90%, respectively. Black font indicates a pre-edited state and red font indicates an editing event to reconstitute a conserved codon in a given reference, respectively. A zero indicates lack of homology or presence of a codon that can not be converted by RNA editing in a given reference. All 11 sites listed were confirmed in *Amborella* *ndhD* cDNA analysis which additionally identified silent editing event ndhDeU688LL and non-silent editing ndhDeU947TI, which is predicted by only 50% of the references (Add. File 1).
