# Supplementary material for: Frequent chloroplast RNA editing in early-branching flowering plants: pilot studies on angiosperm-wide coexistence of editing sites and their nuclear specificity factors
Source: BMC Evol Biol. 2016 Jan 25;16:23. doi: 10.1186/s12862-016-0589-0 (PMC4727281; doi:10.1186/s12862-016-0589-0)
Supplement: Additional file 3: — Phylogenetic tree of angiosperm homologues to Arabidopsis thaliana CRR28. (DOCX 22 kb) [file 12862_2016_589_MOESM3_ESM.docx]

**Additional File 3**

**Additional File 3. Phylogenetic tree of angiosperm homologues to *Arabidopsis thaliana* CRR28**. Genbank identifiers are given for the sequences of protein models deposited in GenBank, WGS indicates *de novo* protein translations from whole genome shotgun genome sequences. The phylogenetic tree was obtained based on alignment position covered by at least of 90% of the sequences (517 positions) with the Maximum Likelihood method using the JTT+Γ+I model as implemented in MEGA [67]. Node support determined from 100 bootstrap resampling replicates is shown where it reaches at least 70%.
