# Supplementary material for: Frequent chloroplast RNA editing in early-branching flowering plants: pilot studies on angiosperm-wide coexistence of editing sites and their nuclear specificity factors
Source: BMC Evol Biol. 2016 Jan 25;16:23. doi: 10.1186/s12862-016-0589-0 (PMC4727281; doi:10.1186/s12862-016-0589-0)
Supplement: Additional file 4: — Phylogenetic tree of angiosperm homologues to Arabidopsis thaliana RARE1. (DOCX 19 kb) [file 12862_2016_589_MOESM4_ESM.docx]

**Additional File 4**

**Additional File 4. Phylogenetic tree of angiosperm homologues to *Arabidopsis thaliana* RARE1**. Genbank identifiers are given for the sequences of protein models deposited in GenBank, WGS indicates *de novo* protein translations from whole genome shotgun genome sequences. The phylogenetic tree was obtained with the Maximum Likelihood method using the JTT+F+Γ+I model as implemented in MEGA [67]. Node support determined from 100 bootstrap resampling replicates is shown where at least 70%.
