# Supplementary material for: Frequent chloroplast RNA editing in early-branching flowering plants: pilot studies on angiosperm-wide coexistence of editing sites and their nuclear specificity factors
Source: BMC Evol Biol. 2016 Jan 25;16:23. doi: 10.1186/s12862-016-0589-0 (PMC4727281; doi:10.1186/s12862-016-0589-0)
Supplement: Additional file 5: — Chloroplast primer sequences. (DOCX 34 kb) [file 12862_2016_589_MOESM5_ESM.docx]

**Chloroplast primer sequences**

| **Amplicon/primer names** | **Sequences** |
| --- | --- |
| accD-up-v2  accD-do-v2 | ATGGAAARATGKKGGTTCAATTCGATG  GAAACCTGTGAGCTGAAATAACTC |
| atpA-up-v2  atpA-do-v2 | ATGGTAACCATTCGAGCTGACGAA  AGMTATGATTTCYTGRAAYTGAGG |
| atpB-up-v2  atpB-do-v2 | CAAATCATTGGTCCGGTACTGGATGT  TTCTTTAATTTGCTCTCTACTTCTAAGTT |
| atpF_up  atpF_do | CGGGTTKAATACCGATATTTTWGC  ATCRGTTATTTCTTTCATGG |
| atpI-up  atpI-do | GAAGTMGGCCARCAYTTVTATTGG  ATGATGRCCYTCCATDGAYTCRCC |
| ccsA_up  ccsA_do | GACTTTCTTATGATATTCG  TAGATTGACCCCAAAATAGC |
| cemA-up  cemA-do | YATYTATMGTNYTTYTGCCYTGG  ATSATAAATNAMTMCAAGYGAMGG |
| clpP_up  clpP_do | KATCCTAATCAACCGACTT  CGCTACRAGATCAACAATGC |
| ndhA-up  ndhA-angio-do | AAMGARGTCTATGGNMTNMTATGG  TTAYAGYGAAAMAAGTTGG |
| ndhCKJ-up  ndhCKJ-do | ATGTTYYTGMTTYAYGAATATGA  TRAGCRTCYTGTATYTCATARAA |
| psaC_ndhD_up  psaC_ndhD_do | CATTCAGTAAAKATTTATGATAC  CCCATTTTTAATAAGATTCC |
| ndhB-Atr-up  ndhB-Atr-end | CTTTCATTTGCTTCTCTTCC  TTAAAAGAAGGTATCCTGAGC |
| ndhD_up2  ndhD_do2 | TACATACATGGTTACCAGA  GAACRAAATCRGGATAAATACC |
| ndhG_up  ndhE_do | TGGATTTRCCTGGACCAATACATG  TGATTGATTGATACGAGTGG |
| ndhF_up1  ndhF_do1 | GGAACRTACATATCAATATGC  CATMCGAAACATATAAAATGC |
| ndhF_up2  ndhF_do2 | CCAGATAARAGTCARAAYATGG  CCATTNGTRAYTCCATCAA |
| ndhG_up  ndhG_do | TGGATTTRCCTGGACCAATACATG  TTATVGAAATGAGTTCAAATGG |
| ndhH_ndhA_up  ndhH-angio-do | GATAGTMAATATGGGTCCTC  CATTCTTTTAACTARCTGAGG |
| ndhI-v2-up  ndhI-v3-Atr-do | ATGTTCCCTATGGTGACTGG  GCTATTTTTGTTTGATTCG |
| petA_up  petA_do | GRTAAAGGAASAGATGACTCG  TGTGCCAGAATAACAGATGC |
| petB_angio_up  petB_angio_do | TGTGACTTGTTATAATTGATCC  TACGGCAGTAAGAAGAGGTA |
| petD-angio-up  petD-angio-do | ATGGGARTAACAAAGAAACC  TGGTWTTTCAYRGTTGAATC |
| petLG-up  petLG-do | CATATYTCATTTAGCTCCTTCATG  TYAAADRTCCAACTGATCNCCRCG |
| petN-up  petN-do | GCTTCCCGATTTCACTCTAA  TTTCGAAATATGAACACGGG |
| psaA-up  psaA-do | TCTTTTCCTGATAGCGGGTC  CTGAATTACCTGGGATGCCT |
| psaB_rps14_up  psaB_rps14_do | GTATAGAACTAACTTCGGGA  CGGATAGCCCAAAGTCTCG |
| psaI-angio-up  psaI-angio-do | ATTTTTGTGCCKTTARTRGG  MAATCTTGTTTTTTTGAAC |
| psbA_up  psbA_do | CGAAAGCGCAAGYYTATGG  GCATAACTTCCATACCAAGG |
| psbB-up  psbB-do | ATGGGTTTGCCTTGGTATCG  TACCACATAGTTCCAGCAACG |
| psbC-angio-up  psbC-angio-do | ATGAAAACCTTATATTCCCTG  TCAGTTAAGAGGRGTCATGG |
| psbEFLJ-up  psbEFLJ-do | ATGTCTGGAAGCACRGGAGAACG  TAYAGRGAYGAWCCYAANCCNGAATA |
| psbI-up  psbI-do | TGCTTACTCTCAAACTGTTC  TTATTCGTCACGCCCAGG |
| psbK-up  psbK-v2-do | TACAAGAAYGAAAYGCTTGKTATG  GAAATCTATGTTTCTAAAGC |
| psbN-Atr-up  psbN-Atr-do | GCATTGGTTGAGATCTGTTGACT  TGTTGAGAGGGTTGCCCAAA |
| psbZ-angio-up  psbZ-angio-do | ATGAYTATTGCTTTCCAAT  TCAAGAGATGAGAGAATTAAG |
| rbcL-up  rbcL-do | ATTCATGAGTTGTAGGGAGG  GGGAACGAACAGGAATCACT |
| rpl16_14_rps8_up  rpl16_14_rps8_do | CGTATGGGTTCGGGRAARGG  RTCYGTCATTATACCTTGAG |
| rpl16-Atr-up  rpl16-Atr-do | ATGCTTAGTGTGTGACTCGTTG  CTCTGGCGACAGTTTCTGG |
| rpl20-Atr-up  rpl20-Atr-do | ACTCGAACTGCTACTCAACAGA  TCATTGGAAATCATGTAAAGGGA |
| rpl22_rps3_up  rpl22_rps3_do | AATATGGGTTTGAACGAAGC  TTCTTCATCTACAAATATCC |
| rpl23_rpl2_up  rpl23_rpl2_do | TTACAGAAAAAAGTATTCGG  TAATTGTCCACCCCTTCCAAG |
| rpoA-up  rpoA-do | ACACYACRGTGGAAGTGYRT  GYCCAATAYCYGTTTKACA |
| rpoB-1-up  rpoB-1-do | ATGCTMSGGGATGGAAATGAGGGAAT  CATTTAGAACTCATTAAAGCTCGATTCGC |
| rpoB-2-up  rpoC1-N-rev | TCTATTGGAGCTTCCCTCATTCC  TGAAAAGTATAGGGTTTTGTCAC |
| rpoC1-up-v2  rpoC1-do-v2 | TATAAACATCAACAACTYCGAATTGG  TCTATTTCTCGATAAAAAGAAATATGACC |
| rpoC2-1-up  rpoC2-1-do | ATGGCAGAACGGGCCRATCTGGT  ATTCATTTGATCTTGATCCTTGTG |
| rpoC2-2-up  rpoC2-2-do | CTCTTACCMAAAACAAGYCATTTATGG  TTTGGAAGACCYTGNGTTATATCACC |
| rpoC2-end-up  rpoC2-end-do | CATTTTCCTGGAGAGACGG  CACGACCTATTCTTTCTGCT |
| rpoB-int-up  rpoB-int-do | ACGGGATTTCTGTTTATACC  ATATCTCCATATACCAGACG |
| rpoC1-int-up  rpoC1-int-do | TCTGTTGATTCTGGGATACG  CTGTAATAATTCGCAAATCCG |
| rps2-up  rps2-do | AAATGGAATCCYARAATGGCNCC  CCYYGTCATATATTTRATCCCGCC |
| rps12-up  rps12-do | ATTAGAAACACAAGACAGCC  TTTTGACCCCATATTGTAGG |
| rps16-up  rps16-do | ATGGTAAAACTTCGTTTRAAACGA  TTMYTTAAATACCTCCGCCTTCTT |
| rps18-Atr-up  rps18-Atr-do | ACCTTTTCGTAAATCCAAGCGA  TCTAGGGAGGGATTCGGTTCT |
| ycf1-1-up  ycf1-1-do | AGAAGGAACCGAGAAGGAGG  CCTCGTCTTGTTCCAAATCG |
| ycf1-2-up  ycf1-2-do | GATTACTCTATAAATGGCG  TCGTTCATTCCGAAATTACC |
| ycf1-3-up  ycf1-3-do | AGGTTTATCGTTGATATGG  TATTTATGTTTCCTTTTCCG |
| ycf1-4-up  ycf1-4-do | TCTTTACTCATACTGGTGCC  TCTCTGATTGTGGTTGTCTT |
| ycf2-1-Atr-up  ycf2-1-Atr-do | GACATCAACTCAAATCCTGG  GAAATGATCTCTGGATTGAT |
| ycf2-2-Atr-up  ycf2-2-Atr-do | TCCCCATTCATAGGTCCGAGA  CATGTTGCCGCCAGAATCAG |
| ycf2-3-up  ycf2-3-do | CATACATGATTGAGTTGCGA  CSTTACATGATTTCTTCTTC |
| ycf2-4-up  ycf2-4-do | ATGGGTTCCAATGCACGAGA  TCATCCGGGAAAAGCCATCT |
| ycf3-up  ycf3-do | AAYGGMAATTTYATHGATAAGAC  CGYYTCGTDATYTTYAACCARTT |
| ycf4-Atr-up  ycf4-Atr-do | TGGATAGAACTTATAACAGG  CGCAGACGGGGAACAGAAGG |
